# *PRNP* D178N-129MM prion disease: Creutzfeldt-Jakob disease-like cortical ribboning without prominent early insomnia

**DOI:** 10.3389/fnins.2026.1950003

**Published:** 2026-09-25

**Authors:** Wanyi Wei, Haitian Nan, Zhongyun Chen, Junjie Li

**Affiliations:** 1 Department of Neurology, Xuanwu Hospital, Capital Medical University, Beijing, China; 2 Department of Neurology, Hebei General Hospital, Shijiazhuang, Hebei, China

**Keywords:** cortical ribboning, diffusion-weighted imaging, fatal familial insomnia, prion disease, *PRNP*, sleep–wake disorders

## Abstract

**Background:**

The *PRNP* p.Asp178Asn (D178N) mutation on a methionine-bearing codon-129 allele is classically associated with fatal familial insomnia (FFI), but some carriers develop a Creutzfeldt-Jakob disease (CJD)-like phenotype.

**Case presentation and findings:**

We report a 59-year-old woman with two months of rapidly progressive cognitive-language decline, caregiver-observed nocturnal sitting-up and sleep talking, weight loss, extensive cortical diffusion-weighted imaging (DWI) ribboning, positive cerebrospinal fluid 14–3-3, and positive multisite skin real-time quaking-induced conversion. Electroencephalography showed diffuse slowing without periodic sharp-wave complexes. *PRNP* sequencing identified D178N with methionine homozygosity at codon 129. A focused literature review identified six previously reported individual D178N-129MM cases with prion-compatible cortical or cortical-striatal DWI abnormalities. Because these reports were selectively ascertained by imaging and used heterogeneous clinical assessments, they were not treated as a clinical series or compared numerically with MRI-negative cohorts. Other D178N-129MM reports show normal DWI in non-insomnia cognitive or motor presentations, objective sleep abnormalities preceding insomnia, and occasional cortical DWI hyperintensity in FFI cohorts.

**Conclusion:**

D178N-129MM disease shows substantial phenotypic and imaging heterogeneity. Marked CJD-like cortical ribboning can occur in an initially non-sleep-dominant presentation and should not exclude an FFI-associated genotype. MRI pattern alone should not be used to infer a specific clinical phenotype; *PRNP* sequencing and objective sleep assessment remain important when clinically appropriate.

## 1 Introduction

Fatal familial insomnia (FFI) is classically associated with *PRNP* p.Asp178Asn (D178N) on a methionine-bearing codon-129 allele (Goldfarb et al., 1992). However, this genotype–phenotype association is not absolute. D178N-129MM disease can present with a genetic Creutzfeldt-Jakob disease (CJD) phenotype (Zarranz et al., 2005), and recent work emphasizes substantial variability even among carriers with the same genotype (Mead et al., 2026). Sleep–wake disturbance and dysautonomia remain characteristic of FFI, but insomnia need not be the first symptom: in a 16-patient FFI cohort, insomnia was the initial symptom in 5 patients, while cortical diffusion-weighted imaging (DWI) hyperintensity was present in 2 patients, including one with codon-129 MM (Kortazar-Zubizarreta et al., 2023). Conversely, D178N-129MM patients with parkinsonism or dementia without prominent insomnia and with normal DWI have been reported (Fukuoka et al., 2018; Park et al., 2021). Cortical ribboning on DWI remains a characteristic imaging marker of CJD and is highly informative in rapidly progressive dementia (Vitali et al., 2011; Manara et al., 2024), but it should not substitute for genetic classification. We describe D178N-129MM prion disease with a cortical cognitive-language syndrome, extensive ribboning, and only subtle early nocturnal manifestations, and provide a focused review of comparable DWI-positive reports with broader contextual literature to emphasize phenotype-imaging overlap.

## 2 Methods

Clinical information was obtained from the medical record with guardian consent. A focused PubMed/MEDLINE search and backward and forward citation tracking were updated through August, 2026. The primary search combined D178N/Asp178Asn and codon-129/FFI/CJD terms with MRI/DWI terms; the exact Boolean strategy and screening framework are provided in Supplementary material (methods). Eligible DWI-positive reports provided individual-level *PRNP* D178N, codon-129 MM, and explicit prion-compatible cortical or cortical-striatal DWI abnormalities. Reports without extractable individual DWI findings, with secondary non-prion causes of diffusion abnormality, or with uncertain duplicate cases were excluded. This focused component was designed to identify imaging analogues of the present patient, not to estimate the prevalence of clinical phenotypes or MRI findings. For broader clinical context, we separately reviewed relevant D178N-129MM reports irrespective of DWI positivity, including individual cases and larger FFI cohorts, without pooling them as a comparator group. No direct numerical group comparisons or inferential tests were performed.

The study was approved by the Ethics Committee of Xuanwu Hospital, Capital Medical University, and was conducted in accordance with the Declaration of Helsinki. Written informed consent for participation and publication was obtained from the patient’s guardian.

## 3 Results

### 3.1 Present case

A 59-year-old woman developed progressive memory impairment and reduced spontaneous speech over two months, followed by mixed aphasia. Her family observed repeated nocturnal sitting-up and sleep talking with morning amnesia, but no prominent refractory insomnia. Depressed mood, reduced oral intake, and an unintentional 5-kg weight loss occurred concurrently. There was no known family history of a similar neurological disorder.

On admission, she was awake but slow to respond, with severe impairment of memory, comprehension, orientation, and calculation. Cranial nerves, strength, tone, reflexes, plantar responses, and coordination were unremarkable. Routine blood tests and basic CSF indices were normal. Infectious, autoimmune encephalitis, paraneoplastic, and demyelinating evaluations were negative.

CSF 14–3-3 was positive, and multisite skin real-time quaking-induced conversion (RT-QuIC), performed as previously described (Chen et al., 2024), was positive. Electroencephalography showed moderate diffuse slowing without periodic sharp-wave complexes. Polysomnography was not obtained; nocturnal events were caregiver-reported. MRI showed extensive bilateral ribbon-like DWI hyperintensity in the frontal, temporal, parietal, and occipital cortices, without dominant thalamic involvement. Corresponding Apparent Diffusion Coefficient (ADC) maps showed low signal in the same cortical regions, confirming true diffusion restriction; corresponding Fluid-Attenuated Inversion Recovery (FLAIR) abnormalities were less conspicuous (Figure 1). Sanger sequencing identified *PRNP* c.532G > A (p.Asp178Asn) with methionine homozygosity at codon 129. The initial impression was CJD; genetic testing established D178N-129MM genetic prion disease with a CJD-like phenotype. At two months after discharge (month 4 from onset), cognition, memory, and language had slightly declined; no prominent insomnia, dysautonomia, or other characteristic FFI manifestations had emerged.

**Figure 1:**
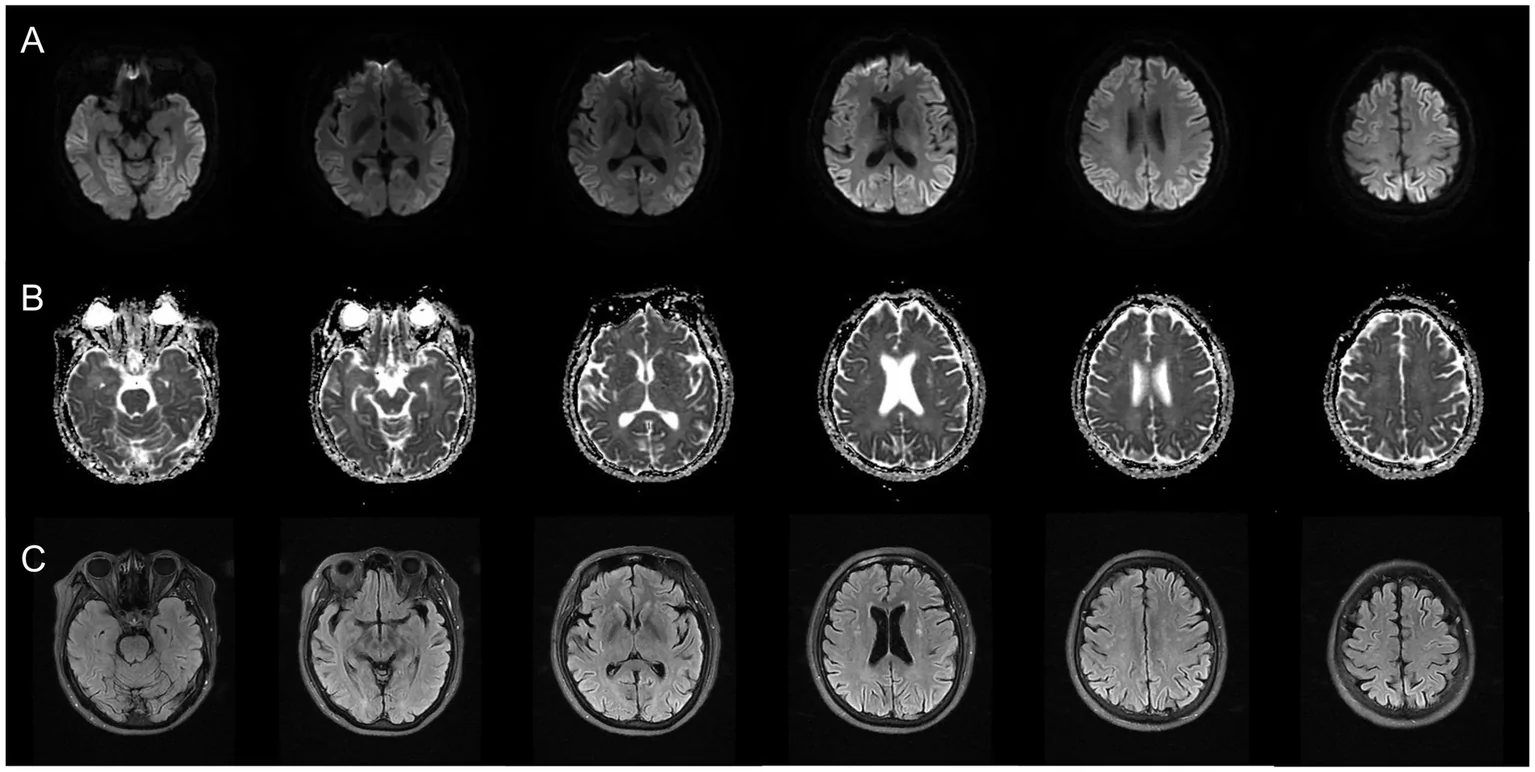
Representative axial diffusion-weighted images **(A)** show extensive bilateral cortical ribbon-like hyperintensity in the frontal, temporal, parietal, and occipital cortices, mimicking a Creutzfeldt-Jakob disease pattern. No dominant thalamic DWI abnormality was evident. Corresponding ADC maps **(B)** show low signal in the same cortical regions, confirming true diffusion restriction. Representative FLAIR images **(C)** show less conspicuous corresponding cortical signal abnormalities.

### 3.2 Reported DWI-positive cases

Six eligible published patients were identified: Choi et al. (2009), Chen et al. (2015), Sun et al. (2015), Huang et al. (2020), and Taweephol et al. (2024). Together with the present patient, seven individual DWI-positive cases were summarized (Table 1). These independently reported cases used heterogeneous clinical and imaging assessments and are therefore not considered a clinical series or a representative D178N-129MM phenotype. Presentations included cognitive, behavioral, language, and motor-predominant syndromes. The sleep descriptions were also heterogeneous: prominent insomnia was documented as absent in some reports, not reported or not emphasized in others, and developed later in several cases; other nocturnal manifestations were sometimes present. All seven had cortical DWI abnormalities, and two also had striatal involvement. CSF 14–3-3 was positive in three of four tested patients, and periodic sharp-wave complexes were present in one of six assessed.

**Table 1:** *PRNP* D178N-129MM cases with cortical or cortical-striatal DWI abnormalities.

Reference	Age/sex	Dominant presentation	Sleep–wake manifestations	DWI findings	Prion biomarkers/EEG
Choi et al. (2009)	67/M	Motor/extrapyramidal; later dementia	Insomnia not reported	Bilateral parietal-occipital cortex	CSF 14–3-3 positive; EEG normal
Chen et al. (2015)	46/F	Early severe dementia; CJD-like	Minor insomnia developed late	Widespread cortex and bilateral striatum	CSF 14–3-3 positive; diffuse slowing
Sun et al. (2015)	51/M	Rapid dementia and behavioral change	No early refractory insomnia; worsened later	Mild right frontal cortex	14–3-3 not reported; diffuse theta-delta slowing
	70/F	Rapid cognitive decline with dysautonomia	Prominent insomnia not reported	Extensive bilateral frontal, temporal, parietal, and occipital cortex	CSF 14–3-3 negative; no PSWC
Huang et al. (2020)	58/F	Corticobasal syndrome evolving to dementia	Insomnia documented absent	Bilateral striatum and medial frontal cortex	Prion biomarkers not reported; PSWC present
Taweephol et al. (2024)	58/F	Rapid cognitive decline and abnormal movements	Insomnia developed later	Anterior cingulate, insular, and orbitofrontal cortices	CSF RT-QuIC negative; mildly elevated t-tau; diffuse dysfunction with sleep–wake disruption
Present case	59/F	Rapid dementia with mixed aphasia	No prominent early insomnia; nocturnal behaviors present	Extensive bilateral frontal, temporal, parietal, and occipital cortex	CSF 14–3-3 positive; skin RT-QuIC positive; diffuse slowing without PSWC

DWI, diffusion-weighted imaging; PSWC, periodic sharp-wave complexes; RT-QuIC, real-time quaking-induced conversion. Sleep entries distinguish documented absence, lack of reporting or lack of prominence, and delayed insomnia where the source data allow. These cases were selected for reported prion-compatible DWI abnormalities and should not be considered a clinical series or a representative D178N-129 MM phenotype. The report by Taweephol et al. (2024) is a conference abstract.

### 3.3 Contextual D178N-129MM reports

Published D178N-129MM observations not selected for DWI positivity illustrate substantial phenotype-imaging overlap. Wu et al. (2017) described a five-patient, single-center retrospective cohort focused on FFI and sleep abnormalities; two patients were siblings, all had marked polysomnographic abnormalities, and MRI was reported as showing no abnormalities, but detailed DWI/ADC characterization was not provided. We therefore use this cohort only as clinical context, not as a DWI-negative reference group. Park et al. (2021) reported two affected sisters with rapidly progressive parkinsonism/dementia, autonomic or sleep-related respiratory/nocturnal manifestations, no insomnia, and normal DWI. Fukuoka et al. (2018) described parkinsonism, dementia, and hallucinations with initially no insomnia or dysautonomia and no diffusion-MRI abnormality; polysomnography was abnormal, and insomnia plus sympathetic autonomic symptoms emerged later. In the 16-patient FFI cohort of Kortazar-Zubizarreta et al. (2023), 12 patients were codon-129 MM, insomnia was the first symptom in only 5/16, and cortical DWI hyperintensity occurred in 2/16, including 1/12 MM patients. A recent longitudinal D178N-129MM FFI report also showed persistently normal T1-weighted, FLAIR, and DWI MRI despite progressive thalamic and cortical metabolic abnormalities on FDG-PET (Chen et al., 2025). These reports were not pooled because their ascertainment, study design, and reporting differed substantially.

## 4 Discussion

The strongest inference from the present case and the literature is phenotypic and imaging heterogeneity within D178N-129MM disease. Our patient had rapidly progressive cortical dysfunction with extensive CJD-like DWI ribboning while prominent refractory insomnia was not an early clinical feature. The selectively ascertained DWI-positive reports show that this imaging pattern can occur across varied clinical presentations, but they cannot establish an association between DWI pattern and sleep phenotype because publication, ascertainment, and reporting were conditioned on the imaging feature of interest. Likewise, caregiver-reported nocturnal behaviors in the present case do not establish occult thalamic sleep pathology in the absence of polysomnography.

The broader literature argues against a simple sleep-dominant/DWI-negative versus cortical/DWI-positive dichotomy. Park et al. (2021) and Fukuoka et al. (2018) demonstrate that normal DWI can coexist with rapidly progressive cognitive or motor syndromes in D178N-129MM disease, including presentations without initial insomnia. Fukuoka et al. further showed objective sleep abnormalities before overt insomnia and dysautonomia, highlighting the difference between subjective clinical insomnia and physiological sleep disruption. Conversely, Kortazar-Zubizarreta et al. (2023) found occasional cortical DWI hyperintensity in a systematically characterized FFI cohort, including a codon-129 MM patient, while insomnia was the first symptom in only a minority. The sleep-focused Wu et al. (2017) cohort remains informative regarding polysomnographic FFI manifestations, but its design, familial clustering, and limited sequence-level MRI reporting preclude its use as a representative DWI-negative comparison group.

This overlap is biologically plausible. Codon 129 is a major determinant of the D178N-related phenotype (Goldfarb et al., 1992), but clinical expression varies substantially among D178N carriers (Zarranz et al., 2005; Mead et al., 2026). Metabolic imaging also demonstrates abnormalities that may precede or exceed structural MRI changes: a longitudinal D178N-129MM case showed normal T1-weighted, FLAIR, and DWI MRI despite thalamic, frontal, and later parietal/striatal hypometabolism (Chen et al., 2025), while PET/MRI studies have identified thalamic subnuclear abnormalities in FFI (Xie et al., 2022). The DWI-positive reports summarized here foreground neocortical and occasional striatal injury because that was the selection criterion; they should not be interpreted as a distinct biological subtype. Whether imaging patterns reflect prion strain, host modifiers, disease stage, or combinations of these factors requires longitudinal multimodal imaging, polysomnography, and neuropathological confirmation.

Multisite skin RT-QuIC can detect prion seeding activity in a peripheral specimen and complement CSF testing (Chen et al., 2024). Seed amplification assays have substantially improved prion-disease diagnosis, but they do not by themselves define the underlying *PRNP* mutation or clinicopathological subtype (Zerr et al., 2026). *PRNP* sequencing therefore remains necessary for etiological classification of suspected genetic prion disease (Mead et al., 2026). Absence of a family history should not preclude testing, because phenotypic variability and incomplete family information may obscure recognition of inherited disease (Zarranz et al., 2005).

For sleep-medicine practice, D178N-129MM disease may include insomnia, sleep–wake disorganization, dysautonomia, weight loss, rapidly progressive cognitive symptoms, and motor manifestations, but their timing and prominence vary (Zhang et al., 2022; Kortazar-Zubizarreta et al., 2023). Caregiver-reported sleep talking, repeated nocturnal behaviors, sleep-related respiratory phenomena, dysautonomia, or unexplained weight loss should therefore prompt a structured sleep history and, when feasible, video-polysomnography rather than reliance on a binary question about insomnia. Conversely, CJD-like ribboning should not exclude an FFI-associated genotype, and normal DWI should not be assumed to identify a sleep-dominant phenotype.

Limitations include the absence of neuropathology in the present patient and the short follow-up, currently limited to month 4 from onset. The literature component is a focused review of DWI-positive analogues rather than a systematic estimate of the full D178N-129MM spectrum; the included reports are small, publication-derived, heterogeneous, and subject to ascertainment and reporting bias, including one conference abstract (Taweephol et al., 2024). Contextual reports were reviewed narratively and were not treated as controls or pooled comparators. Accordingly, no association between DWI pattern and clinical phenotype can be inferred. The principal contribution is the documentation of marked CJD-like cortical DWI ribboning in D178N-129MM disease with an initially non-sleep-dominant presentation.

## 5 Conclusion

D178N-129MM prion disease is clinically and radiologically heterogeneous and can present with extensive CJD-like cortical ribboning despite no prominent early insomnia. This observation might be interpreted as an example of overlap rather than evidence for a distinct DWI-defined phenotype. *PRNP* sequencing should be considered in rapidly progressive cortical syndromes, and subtle nocturnal or autonomic features should prompt objective sleep assessment when feasible.

## Data Availability

The original contributions presented in the study are included in the article/Supplementary material, further inquiries can be directed to the corresponding author.
