# Supplementary material for: PRNP D178N-129MM prion disease: Creutzfeldt-Jakob disease-like cortical ribboning without prominent early insomnia
Source: Front Neurosci. 2026 Sep 25;20:1950003. doi: 10.3389/fnins.2026.1950003 (PMC13649760; doi:10.3389/fnins.2026.1950003)
Supplement: Supplementary file 1 [file Table_1.docx]

Supplementary Material

Supplementary Methods and Table

# 1 Supplementary Methods

PubMed/MEDLINE and backward/forward citation tracking were updated through August, 2026. The focused search used the Boolean strategy: ("D178N" OR "Asp178Asn") AND ("129MM" OR "methionine homozygous" OR "fatal familial insomnia" OR "Creutzfeldt-Jakob disease" OR "genetic prion disease") AND ("MRI" OR "magnetic resonance" OR "DWI" OR "diffusion-weighted imaging" OR "diffusion" OR "cortical ribboning"). Titles/abstracts were screened for D178N disease, and full texts were reviewed when codon-129 status and individual imaging findings could be found.

DWI-positive eligibility required individual-level *PRNP* D178N, codon-129 MM, and an explicit cortical ribbon-like or cortical-striatal DWI abnormality considered compatible with prion disease. Reports without individual DWI detail, with diffusion abnormalities attributed to a secondary non-prion process, or with uncertain duplication were not counted in Part A. This focused selection was intended to identify imaging analogues of the present case rather than define the full D178N-129MM phenotype or estimate the prevalence of DWI abnormalities.

For clinical context, a separate search using the first two concept blocks above without the imaging block, together with citation tracking, was used to identify relevant D178N-129MM reports irrespective of DWI positivity. Particular attention was paid to individual cases and larger cohorts that inform phenotype-imaging overlap (Wu et al., 2017; Fukuoka et al., 2018; Park et al., 2021; Kortazar-Zubizarreta et al., 2023; Chen et al., 2025). This contextual component was not treated as a matched or representative comparator group, and no pooled numerical comparison or inferential analysis was performed. Full reference details are provided in the main manuscript.

Supplementary Table 1. D178N-129MM DWI-positive cases and contextual literature

Part A. DWI-positive D178N-129MM cases

| **Reference/case** | **Age/sex** | **Dominant presentation** | **Early** sleep/insomnia status | **DWI finding** | **CSF 14-3-3** | **PSWC** |
| --- | --- | --- | --- | --- | --- | --- |
| Choi et al., 2009 | 67/M | Motor/extrapyramidal | Insomnia not reported | Parietal-occipital cortical ribbon | Positive | Absent |
| Chen et al., 2015 | 46/F | Cognitive/dementia | Developed later (minor insomnia) | Diffuse cortical and bilateral striatal | Positive | Absent |
| Sun et al., 2015 | 51/M | Cognitive/behavioral | Not prominent early; worsened later | Mild right frontal cortical | NR | Absent |
| Liu et al., 2017 | 70/F | Cognitive/dementia | Prominent insomnia not reported | Diffuse frontal, temporal, parietal, and occipital cortical | Negative | Absent |
| Huang et al., 2020 | 58/F | Corticobasal/motor | Documented absent | Bilateral striatal and medial frontal cortical | NR | Present |
| Taweephol et al., 2024 | 58/F | Cognitive/behavioral | Developed later | Anterior cingulate, insular, and orbitofrontal cortical | NR | NR |
| Present case | 59/F | Cognitive/language | No prominent early insomnia; nocturnal behaviors present | Diffuse frontal, temporal, parietal, and occipital cortical | Positive | Absent |

Part B. Contextual D178N-129MM reports not selected for DWI positivity

| **Reference / design** | **Population / genotype** | **Clinical presentation** | **Sleep phenotype** | **MRI / DWI findings** | **Interpretive role** |
| --- | --- | --- | --- | --- | --- |
| Wu et al., 2017; single-center retrospective sleep-focused cohort | 5 D178N-129MM patients; 2 were siblings | Rapidly progressive dementia with sympathetic and sleep-related manifestations | Sleep disturbance was prominent; PSG markedly abnormal in all 5 | MRI reported no abnormalities; detailed DWI/ADC characterization not provided | Clinical PSG context only; not a DWI-negative reference cohort |
| Fukuoka et al., 2018; individual case | 58/M; D178N-129MM | Parkinsonism, dementia, visual hallucinations | Initially no insomnia or dysautonomia; PSG abnormal; insomnia and sympathetic autonomic symptoms developed later | Slight diffuse cortical atrophy on T1; diffusion MRI showed no abnormal findings | Demonstrates non-insomnia onset with normal diffusion imaging and later FFI features |
| Park et al., 2021; family report | Two affected sisters; D178N-129MM | Rapidly progressive parkinsonism and dementia with autonomic/respiratory manifestations | No insomnia; dream enactment, snoring/stridor or sleep apnea reported | DWI normal; FLAIR showed mild leukoaraiosis in one and no definite abnormality in the other | Demonstrates normal DWI despite non-sleep-dominant cognitive/motor presentation |
| Kortazar-Zubizarreta et al., 2023; regional FFI cohort | 16 FFI patients; 12 were 129MM | Broad FFI spectrum; cognitive/amnestic deficits common | Insomnia first symptom in 5/16; later clinical or PSG sleep abnormality in nearly all | Cortical DWI hyperintensity in 2/16, including 1/12 MM; no basal-ganglia DWI hyperintensity | Shows overlap: insomnia often not initial and cortical DWI abnormalities can occur in FFI |
| Chen et al., 2025; longitudinal case | 42/F; D178N-129MM | Sleep disorder and autonomic dysfunction | Sleep/autonomic-dominant presentation | T1, FLAIR and DWI normal at baseline and 3.5-month follow-up; progressive thalamic/cortical/striatal FDG-PET hypometabolism | Shows metabolic abnormalities despite persistently unremarkable structural/diffusion MRI |

Part C. Interpretive note

The DWI-positive cases in Part A and the contextual reports in Part B were identified through different study designs and ascertainment routes. They were therefore not pooled or numerically compared, and no prevalence estimate or phenotype-imaging association was calculated.

Part D. Reports screened but not counted as additional DWI-positive cases

| **Report** | **Reason for exclusion or handling** |
| --- | --- |
| Guerreiro et al., 2009 | D178N-cis-129M dementia without insomnia, but no extractable individual DWI description. |
| Chen et al., 2018 | Seven D178N-129MM patients; one CJD-pattern MRI at cohort level. Individual detail was insufficient to establish independence from earlier reports. |
| Froböse et al., 2014 | DWI abnormality occurred with posterior reversible encephalopathy syndrome and was not a prion-pattern cortical ribbon. |
| Lu et al., 2017 | Diffuse abnormalities were attributed to coexisting CADASIL rather than prion-pattern cortical ribboning. |
| Shi et al., 2021 | National surveillance data provided aggregate MRI counts without case-level information adequate for deduplication. |
| Chen et al., 2025 | Included in Part B as contextual longitudinal imaging evidence; not counted in Part A because T1, FLAIR, and DWI remained normal. |

Definitions: “DWI-positive” required an explicit cortical ribbon-like or cortical-striatal diffusion abnormality considered compatible with prion disease. Sleep/insomnia status was transcribed as specifically as source data allowed: “documented absent,” “not reported,” “not prominent early,” “developed later,” or other nocturnal/sleep manifestations. A missing or unreported insomnia description was not treated as documented absence. NR, not reported; PSWC, periodic sharp-wave complexes; PSG, polysomnography.

References

Choi, B. Y., Kim, S. Y., Seo, S. Y., An, S. S. A., Kim, S. Y., Park, S. E., et al. (2009). Mutations at codons 178, 200-129, and 232 contributed to the inherited prion diseases in Korean patients. BMC Infect. Dis. 9, 132. doi: 10.1186/1471-2334-9-132.

Chen, S., Guan, M., Shang, J. K., He, S., Zhang, M. L., Ma, M. M., et al. (2015). Reduced cerebral blood flow in genetic prion disease with *PRNP* D178N-129M mutation: an arterial spin labeling MRI study. *J. Clin. Neurosci.* 22, 204–206. doi: 10.1016/j.jocn.2014.05.040.

Sun, L., Li, X., Lin, X., Yan, F., Chen, K., and Xiao, S. (2015). Familial fatal insomnia with atypical clinical features in a patient with D178N mutation and homozygosity for Met at codon 129 of the prion protein gene. *Prion* 9, 228–235. doi: 10.1080/19336896.2015.1054601.

Liu, L., Li, C., Yang, Q., Zhang, W., Liu, Y., and Zhu, H. (2017). Clinical and neuroimaging features of a Chinese patient with fatal familial insomnia. *Sleep Med.* 32, 280–281. doi: 10.1016/j.sleep.2016.12.001.

Huang, Y., Ma, J., Morales, R., and Tang, H. (2020). Corticobasal manifestations of Creutzfeldt-Jakob disease with D178N-homozygous 129M genotype. *Prion* 14, 232–237. doi: 10.1080/19336896.2020.1812367.

Taweephol, T., Pongpitakmetha, T., Hemachudha, P., and Thanapornsangsuth, P. (2024). Atypical case of prion disease with D178N-homozygous 129M genotype. *Neurology* 103 (7 Suppl. 1), S52–S53. doi: 10.1212/01.wnl.0001051320.64658.bc.

Wu, L., Lu, H., Wang, X., Liu, J., Huang, C., Ye, J., et al. (2017). Clinical features and sleep analysis of Chinese patients with fatal familial insomnia. *Sci. Rep.* 7, 3625. doi: 10.1038/s41598-017-03817-3.

Fukuoka, T., Nakazato, Y., Yamamoto, M., Miyake, A., Mitsufuji, T., and Yamamoto, T. (2018). Fatal familial insomnia initially developing parkinsonism mimicking dementia with Lewy bodies. Intern. Med. 57, 2719–2722. doi: 10.2169/internalmedicine.0573-17.

Park, K. W., Lee, S. H., Hwang, Y. S., Kim, K. W., Park, K. Y., Kim, M.-J., et al. (2021). Rapidly progressive parkinsonism and dementia with no insomnia due to the PRNP D178N mutation. J. Clin. Neurol. 17, 579–581. doi: 10.3988/jcn.2021.17.4.579.

Kortazar-Zubizarreta, I., Eraña, H., Pereda, A., Charco, J. M., Manero-Azua, A., Ruiz-Onandi, R., et al. (2023). Analysis of a large case series of fatal familial insomnia to determine tests with the highest diagnostic value. J. Neuropathol. Exp. Neurol. 82, 169–179. doi: 10.1093/jnen/nlac113.

Chen, Z., Wu, L., Yan, S., and Lu, J. (2025). Follow-up multimodal changes on PET/MRI in fatal familial insomnia patient: a case report. *BMC Neurol.* 25, 301. doi: 10.1186/s12883-025-04315-0.

Guerreiro, R. J., Vaskov, T., Crews, C., Singleton, A., and Hardy, J. (2009). A case of dementia with *PRNP* D178Ncis-129M and no insomnia. *Alzheimer Dis. Assoc. Disord.* 23, 415–417. doi: 10.1097/WAD.0b013e3181ae3a76.

Chen, S., He, S., Shi, X. H., Shen, X. J., Liang, K. K., Zhao, J. H., et al. (2018). The clinical features in Chinese patients with PRNP D178N mutation. Acta Neurol. Scand. 138, 151–155. doi: 10.1111/ane.12924.

Froböse, T., Förstl, H., and Förschler, A. (2014). Fatal familial insomnia (FFI) complicated by posterior reversible encephalopathy syndrome (PRES). *Clin. Neuroradiol.* 24, 289–291. doi: 10.1007/s00062-013-0243-9.

Lu, T., Pan, Y., Peng, L., Qin, F., Sun, X., Lu, Z., et al. (2017). Fatal familial insomnia with abnormal signals on routine MRI: a case report and literature review. *BMC Neurol.* 17, 104. doi: 10.1186/s12883-017-0886-2.

Shi, Q., Chen, C., Xiao, K., Zhou, W., Gao, L. P., Chen, D. D., et al. (2021). Genetic prion disease: insight from the features and experience of China National Surveillance for Creutzfeldt-Jakob Disease. *Neurosci. Bull.* 37, 1570–1582. doi: 10.1007/s12264-021-00764-y.
